# SERCA directs cell migration and branching across species and germ layers

**DOI:** 10.1242/bio.026039

**Published:** 2017-08-17

**Authors:** Danielle V. Bower, Nick Lansdale, Sonia Navarro, Thai V. Truong, Dan J. Bower, Neil C. Featherstone, Marilyn G. Connell, Denise Al Alam, Mark R. Frey, Le A. Trinh, G. Esteban Fernandez, David Warburton, Scott E. Fraser, Daimark Bennett, Edwin C. Jesudason

**Affiliations:** 1 Division of Biological Sciences, California Institute of Technology, Pasadena, CA 91125, USA; 2 The Saban Research Institute, Children's Hospital Los Angeles, Los Angeles, CA 90027, USA; 3 Department of Biochemistry & Centre for Cell Imaging, Institute of Integrative Biology, University of Liverpool, Liverpool L69 7ZB, UK; 4 Division of Child Health, Institute of Translational Medicine, University of Liverpool, Liverpool L12 2AP, UK; 5 Craniofacial Biology, Herman Ostrow School of Dentistry, University of Southern California, Los Angeles, CA 90089, USA; 6 Biological Sciences and Biomedical Engineering, University of Southern California, Los Angeles, CA 90089, USA; 7 Biological Sciences and Molecular and Computational Biology, Translational Imaging Center, University of Southern California, Los Angeles, CA 90089, USA; 8 Center for Space and Habitability, University of Bern, 3012 Bern, Switzerland; 9 NHS Lothian, Edinburgh, EH14 1TY, UK; 10 Department of Diagnostic, Interventional and Pediatric Radiology, Inselspital, Bern University Hospital, University of Bern, 3010 Bern, Switzerland, and the Department of Biomedical Research, University of Bern, 3008 Bern, Switzerland

**Keywords:** Branching morphogenesis, Cell migration, SERCA, Calcium dynamics

## Abstract

Branching morphogenesis underlies organogenesis in vertebrates and invertebrates, yet is incompletely understood. Here, we show that the sarco-endoplasmic reticulum Ca^2+^ reuptake pump (SERCA) directs budding across germ layers and species. Clonal knockdown demonstrated a cell-autonomous role for SERCA in *Drosophila* air sac budding. Live imaging of *Drosophila* tracheogenesis revealed elevated Ca^2+^ levels in migratory tip cells as they form branches. SERCA blockade abolished this Ca^2+^ differential, aborting both cell migration and new branching. Activating protein kinase C (PKC) rescued Ca^2+^ in tip cells and restored cell migration and branching. Likewise, inhibiting SERCA abolished mammalian epithelial budding, PKC activation rescued budding, while morphogens did not. Mesoderm (zebrafish angiogenesis) and ectoderm (*Drosophila* nervous system) behaved similarly, suggesting a conserved requirement for cell-autonomous Ca^2+^ signaling, established by SERCA, in iterative budding.

## Abstract (precis)

**Summary:** Dynamic imaging of living embryos demonstrates that SERCA is a conserved regulator that controls cell migration and branching across species and germ layers, and PKC activation can rescue SERCA inhibition. This article has an associated First Person interview with the first author of the paper as part of the supplementary information.

## INTRODUCTION

Branching morphogenesis through repetitive budding offers a powerful means to build complex structures without the information costs of separately encoding each branch (Hogan, 1999); however, such efficiencies must be balanced carefully, as they also facilitate pathological branching, such as tumor angiogenesis and proliferative retinopathy. This mandates a mechanistic understanding of iterative budding and its regulation, yet bud iteration has not been explained in terms of its fundamental cellular behaviors, such as cell-shape change, migration, and proliferation (Fig. S1). Shape change allows single cells to branch (e.g. axons) or create basic tubes by self-canalizing or fusing (e.g. *Drosophila* trachea), migration permits cells to rearrange themselves to form tubal networks (e.g. zebrafish intersomitic vasculature, *Drosophila* trachea), and proliferation permits the scaling up needed to form more extensive branched structures in larger organisms (e.g. human lung) (Affolter et al., 2009). A conserved ‘master routine’ (Metzger et al., 2008) that directs the timing and implementation of specialized branching sub-modules would permit the evolution of complex specialized branching structures while preserving a robust regulatory foundation.

In animals, growth factors have been proposed to play key roles, acting as morphogens that direct repetitive budding and integrate broader influences such as oxygen (Jarecki et al., 1999). Tissue-specific growth factor ‘morphogen clocks’ have been proposed to explain the stereotypic pattern of budding (Metzger et al., 2008; Scott et al., 2010). However, extensive investigations of growth factors have yet to define a master program governing branch iteration. We adopted an alternative approach, based on two lines of reasoning. First, the cell behaviors used for budding (shape change, migration and proliferation) each have antecedents in unicellular organisms which are more basal than metazoans and their morphogens, suggesting that the conserved programs controlling budding are unlikely to rely upon morphogens. Secondly, multicellular morphogenesis requires a robust balance between reliability of signal transmission and flexibility to modulate the signal. Morphogen clocks may be suboptimal for achieving this balance given the substantial variation in gene expression that can exist even between identical adjacent cells (Elowitz et al., 2002). In contrast, cellular Ca^2+^ signaling has been shown by modeling and empirical studies to offer both signal reliability and flexibility in the face of variable protein expression (Abell et al., 2011). Furthermore, Ca^2+^ cycling can regulate budding, whether unicellular or multicellular, in fungi and plants (Torralba and Heath, 2001; Trewavas and Knight, 1994). In animals, repetitive Ca^2+^ waves occur in varied aspects of development, including during organogenesis of the mammalian lung. Live imaging with Ca^2+^ sensitive fluorophores shows periodic propagating Ca^2+^ waves in normally developing vertebrate lungs. Additionally, these waves are abnormal during the reduced branching in hypoplastic lungs (Featherstone et al., 2005; Featherstone et al., 2006).

Given the diverse settings in which Ca^2+^ waves appear correlated with budding and branching, we have tested their causal roles. Repetitive Ca^2+^ waves depend critically on SERCAs (sarco-endoplasmic reticulum Ca^2+^ reuptake pumps). These are the P-type ATPases that return cytosolic Ca^2+^ to the endoplasmic reticulum, and regulate cardiac periodicity and contractility (Wu et al., 1995; Sanyal et al., 2006). Lung Ca^2+^ waves require SERCA and are abolished by the specific inhibitor, cyclopiazonic acid (CPA) (Featherstone et al., 2005; Seidler et al., 1989). We hypothesized that SERCA controls Ca^2+^ activity to regulate the ‘spatial periodicity’ of branching, and thus may serve as a conserved central organizer of iterative branching. To investigate this possibility, we manipulated SERCA function during budding of diverse systems: *Drosophila* airway and nerves, zebrafish intersegmental vessels, and mammalian lung. The results demonstrate that SERCA controls repetitive budding by establishing asymmetric Ca^2+^ levels at branch sites to direct cell migration, and that key morphogens (FGF, EGF) require SERCA in order to operate optimally.

## RESULTS

### Budding requires SERCA cell-autonomously for normal epithelial migration and proliferation

**Fig. 1.**
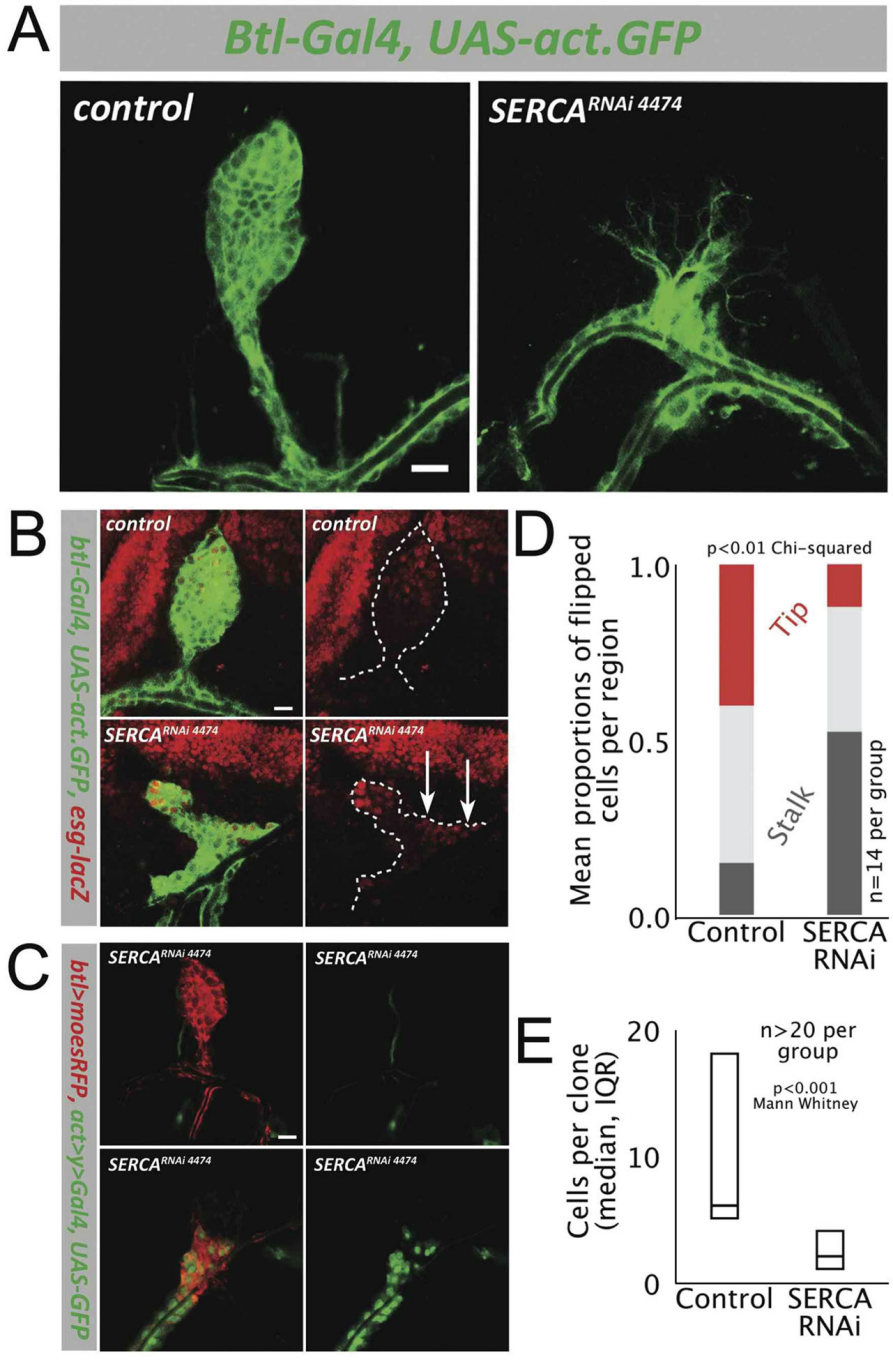
**SERCA inhibition disrupts *Drosophila* air sac via cell-autonomous defects in epithelial migration and proliferation.** (A) *serca* RNAi disrupts airway morphogenesis*.* Wild-type air sac (left), with GFP-labeled respiratory epithelium. *serca* RNAi, driven by *breathless* in respiratory epithelium, results in a stunted (right) or absent air sac. Scale bar: 25 μm. (Also see Fig. S2.) (B) *serca* RNAi alters the position of cells expressing distal marker, *escargot* (red), which normally are positioned only at the air sac tip (top panels). In *serca* RNAi mutants (bottom), *escargot*-expressing cells are retained also in the malformed air sac stalk (arrows, right). *breathless-*dependent GFP expression identifies the wild-type air sac (top row, left) and abnormal *serca* RNAi epithelial air sac (bottom, left). Scale bar: 20 μm. (C) *serca-*deficient cells are necessary within the air sac to disrupt budding*.* FLP-recombinase to generate *serca* RNAi clones (green) shows that if no such clones are induced in the air sac it forms normally (top, epithelium in red), whereas when flipped cells populate the air sac, it fails to develop properly (bottom). Scale bar: 25 μm. (D) Flip-out *serca* RNAi cells have a cell-autonomous migration defect*.* Plot shows proportions of flipped cells localized to the tip, middle and stalk thirds of the air sac for control and *serca* RNAi clones. *serca* RNAi cells are relatively excluded from the air sac tip and restricted more to the stalk. (E) Flip-out *serca* RNAi cells have a cell-autonomous proliferation defect*.* Plot of number of cells per contiguous group of flipped cells for control and *serca* RNAi clones. Median (interquartile range) of phosphohistone H3 (PH3)-positive cells per air sac: 2.5 (1-4) control vs 0 (0-1) *serca* RNAi (*n*=18 per group; *P*<0.001, Mann–Whitney).

RNAi knockdown of *serca* in the budding *Drosophila* air sac epithelium was used to examine its functions *in vivo*. The single *serca* gene in *Drosophila* makes RNAi knockdown simpler than in the mammalian lung, which has three *serca* genes (Klämbt et al., 1992). *Serca* mRNA expression and protein function were diminished in the air sac by the first instar larval stage (Fig. S2A,B). Larval air sacs showed absent or severely stunted buds (Fig. 1A; Fig. S2C) and reduced proliferation. The expression of *escargot*, a migration-related transcription factor (Tanaka-Matakatsu et al., 1996) normally expressed in cells of the distal air sac, instead was expressed within cells positioned in the proximal air sac (Fig. 1B, arrows). This suggests that cell differentiation proceeded normally, and the cells which should populate the air sac tip still expressed *escargot*, however they failed to migrate distally and instead were retained within the air sac stalk.

A cell-autonomous requirement for epithelial expression of *serca* was demonstrated by generating labeled, random ‘flip-out’ clones in which *serca* is absent, by using heat-shock induced FLP-recombinase (Harrison and Perrimon, 1993). Air sac development was disrupted when *serca* RNAi clones arose within the air sac, but was unperturbed when they arose outside it (Fig. 1C). Thus, expression of *serca* is required within the air sac epithelial cells for proper formation of the air sac, and adjacent normal cells are unable to compensate for loss of epithelial *serca*. Only 12% of *serca* RNAi cells reached the air sac tip (distal third) compared to 40% of GFP-labeled wild-type epithelial cells; conversely, 52% of *serca* RNAi cells remained within the proximal third (the stalk) of the air sac, compared to 15% of control ‘flipped’ cells (Fig. 1D). The clones of *serca* RNAi cells contained fewer cells than wild-type clones (Fig. 1E). Rates of apoptosis were negligible in both cases (Fig. S3). This does not exclude the possibility that apoptosis could have occurred at an earlier stage, however these results suggest that *serca* deficiency disrupts budding principally via cell-autonomous defects in epithelial migration and proliferation, which remain uncompensated by adjacent wild-type cells.

### Budding requires SERCA to control cell migration, irrespective of proliferation

**Fig. 2.**
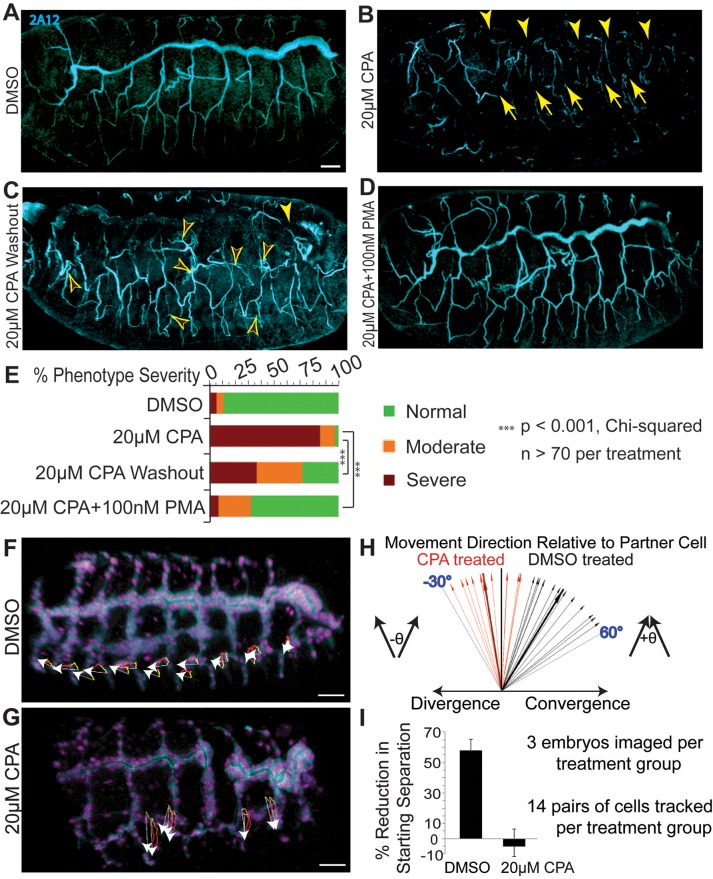
**SERCA regulates cell migration to control budding, even in the absence of proliferation.** (A-E) SERCA blockade reversibly disrupts *Drosophila* tracheogenesis and PMA rescues this*.* Whole mount *Drosophila* embryos at stage 15-16 are viewed from the lateral aspect, anterior left, and the 2A12 antibody stains for tracheal lumen protein following the indicated treatments. (A) DMSO-treated controls display orderly tracheal branches. (B) 20 µM CPA disrupts branching, resulting in gaps in the dorsal trunk (arrowheads) and subsidiary branches (arrows). (C) CPA washout at stage 12 results in fewer breaks (solid arrowhead) and undulating branches with extended sprouts (open arrowheads). (D) PMA with CPA rescues tracheal budding defects. Occasionally, an embryo treated with CPA+PMA or one from which CPA was washed out exhibits a phenotype of excess tracheal cell migration (see Movie 8). (E) Severity of phenotypes was scored for each treatment. Both washout and PMA rescue versus CPA alone significantly reduce the proportions of embryos in higher severity groups (*P*<0.001; Chi-squared; *n*>70 per treatment). (F-I) Live imaging shows SERCA is necessary for airway cells to converge during completion of *Drosophila* tracheogenesis. (F-G) The trajectories (white arrows) and yellow-to-red migration paths are shown for individual cells that form the tracheal lateral trunk from stage 14 to early 16. The displacements shown represent cell movement over 100 min. (F) Cells from adjacent segments in DMSO-treated wild-type embryos converge. (G) During SERCA blockade, tracheal cells lack active migration and slightly diverge as the embryo develops. (H) For pairs of tracheal cells in adjacent segments, the direction of travel of one cell relative to the other was calculated, and a vector was plotted for each pair to compare the movements of all pairs from each treatment together, demonstrating the angles of convergence (controls) or divergence (CPA-treatment) of these cells. (I) The plot shows mean and standard deviation of the convergence, or % reduction in starting separation, of pairs of adjacent cells. Controls reduce their starting separation 58%, while CPA treatment blocks active migration and the cells diverge (the mean is negative). Scale bars: 25 µm throughout.

Branching of the *Drosophila* trachea proceeds without cell proliferation (Samakovlis et al., 1996), and thus serves as a useful model system to study effects of cell migration on branching independently of cell proliferation. RNAi knockdown is ineffective at early embryonic stages, and until late stages, stores of maternal protein result in normal levels of SERCA and of intracellular Ca^2+^ (Fig. S4). CPA inhibition (Seidler et al., 1989) of SERCA protein function disrupted budding, resulting in breaks in the tracheal network that were reversible on washout (Fig. 2A-C; Fig. S5). Ca^2+^-dependent protein kinase Cs (PKCs) enhance SERCA function (Usachev et al., 2006). PKC activation using the agonist, phorbol myristate acetate (PMA), rescued the budding defects induced by SERCA inhibition (Fig. 2D,E; Fig. S5). No cell death was detected to account for these observed gaps (Fig. S5F).

Live imaging of airway cells in *Drosophila* embryos revealed that SERCA's impact on budding occurs instead via effects on airway cell migration. Tracking of airway cells showed how the lateral tracheal branches of *Drosophila* form by convergent cell migration over a few hours (Movie 1). Airway cells converged from adjacent segments of the embryo, reducing their starting separation by nearly 60%, despite the underlying increase in spacing between tracheal segments (Fig. 2F,H,I). This convergent migration failed when SERCA was inhibited; instead, as the embryo grew, the separation of neighboring cells increased by about 5% (Fig. 2G,H,I; Movie 2).

### SERCA directs cell migration at the budding tip by keeping Ca^2+^ levels higher in the leading cell

**Fig. 3.**
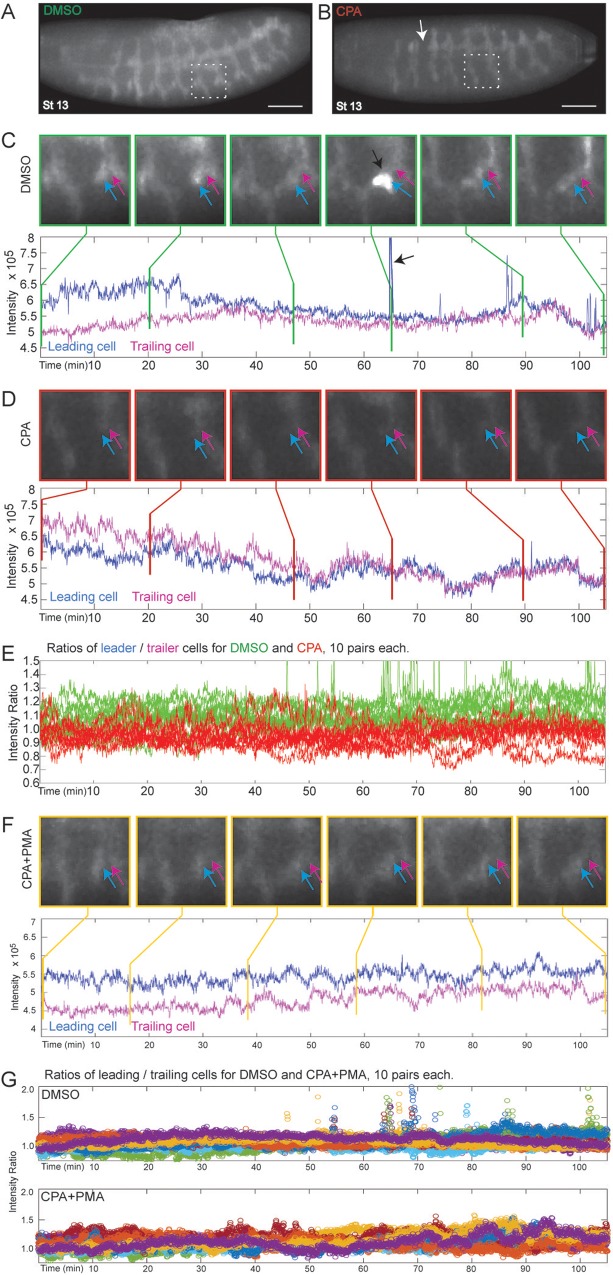
**SERCA regulates cell migration and budding by maintaining higher Ca^2+^ levels in ‘leader’ versus ‘trailer’ cells.** (A,B) *Btl:GCamp3* embryos treated with DMSO, CPA, or CPA+PMA were imaged between stage 13 and 16, and images were reconstructed to analyze the Ca^2+^ levels in tracheal cells. Shown are 3D reconstructed images of stage 13 embryos treated with (A) DMSO or (B) CPA (arrow indicates discontinuous trunk). Insets mark segments tracked in (C) and (D). Scale bars: 50 µm. (C-D,F) Inset images of tracked cells from DMSO- (C), CPA- (D), and CPA+PMA-treated (F) embryos. ‘Leader’ cells (blue arrow) that migrate from adjacent segments and fuse to form the lateral trunk and ‘trailer’ cells (magenta arrow) behind them were tracked over the time course and their Ca^2+^ levels quantified and plotted below the panel of images. Vertical lines mark the time points corresponding to each image. (C) In controls, there is a Ca^2+^ level differential whereby ‘leader’ cells have consistently higher Ca^2+^ levels than ‘trailers’, particularly early when the cells are migrating. After fusion, ‘leaders’ periodically display surges of Ca^2+^ (black arrows; also green spikes in E and spikes in top panel of G). (D) In CPA-treated embryos, migration is lost so the lateral trunk remains discontinuous, and ‘leaders’ have lower Ca^2+^ levels than ‘trailers’ during the time they should be migrating. Thus the Ca^2+^ level differential is reversed. (F) CPA+PMA co-treatment reinstates the higher Ca^2+^ level in ‘leaders’ and rescues migration. (E,G) For each treatment, the ratio of intensities of ten pairs of cells (leader/trailer) was plotted. (E) The DMSO ratios (green) average >1. SERCA inhibition (red, ratio <1) inverts this. (G) The DMSO- and CPA+PMA-treated embryos overlap (ratios >1), so each was plotted separately with different colors for individual ratios. DMSO-treated embryos show Ca^2+^ spikes following trunk fusion. While CPA+PMA restores branching, Ca^2+^ spikes are absent or only seen much later (Movie 5).

Live Ca^2+^ imaging during *Drosophila* tracheogenesis was performed using the GCaMP3 Ca^2+^ indicator, expressed exclusively in tracheal cells (*Btl:GCaMP3*) (Fig. 3A,B). Two-photon light-sheet microscopy was used to visualize the complete tracheal network on one side of the living embryo in 4D, with a time resolution of 3 seconds (Truong et al., 2011). During the formation of the lateral trunk, the leading cells that migrate to fuse with counterparts from neighboring segments (‘leaders’) showed high levels of Ca^2+^ (Fig. 3C). The imaging revealed a lower level of Ca^2+^ in those cells trailing just behind them (‘trailers’), resulting in a Ca^2+^ differential between leaders and trailers. Fig. 3 shows the cells and a graph of the quantified Ca^2+^ level intensities in leader (blue) and trailer (magenta) cells. See Movies 3 and 9 to visualize the Ca^2+^ intensity levels of tracheal cells in the live embryos over time.

Inhibition of SERCA with CPA elevated the overall levels of cytoplasmic Ca^2+^ (Fig. 3D; Fig. S6) and abolished the Ca^2+^ differential between leaders and trailers in many segments of each embryo. In the segments in which the Ca^2+^ differential was lost or reversed (Fig. 3D; Movies 4 and 10), migration failed and the tracheal trunk was disrupted. The loss of the Ca^2+^ differential was apparent by examining the ratios of Ca^2+^ level between leaders and trailers (Fig. 3E). In segments where the Ca^2+^ differential persisted, migration continued and the trunk formed (Movie 4). These findings are consistent with the cell-autonomous nature of SERCA activity, whereby the cells that take up minimal SERCA inhibitor continue to migrate normally, and only those cells that are sufficiently inhibited fail to migrate. PMA not only rescued cell migration and budding from the effects of SERCA blockade, but also normalized overall Ca^2+^ levels, and re-instated the Ca^2+^ differential between leader and trailer cells (Fig. 3F,G; Fig. S6, Movies 5 and 11).

**Fig. 4.**
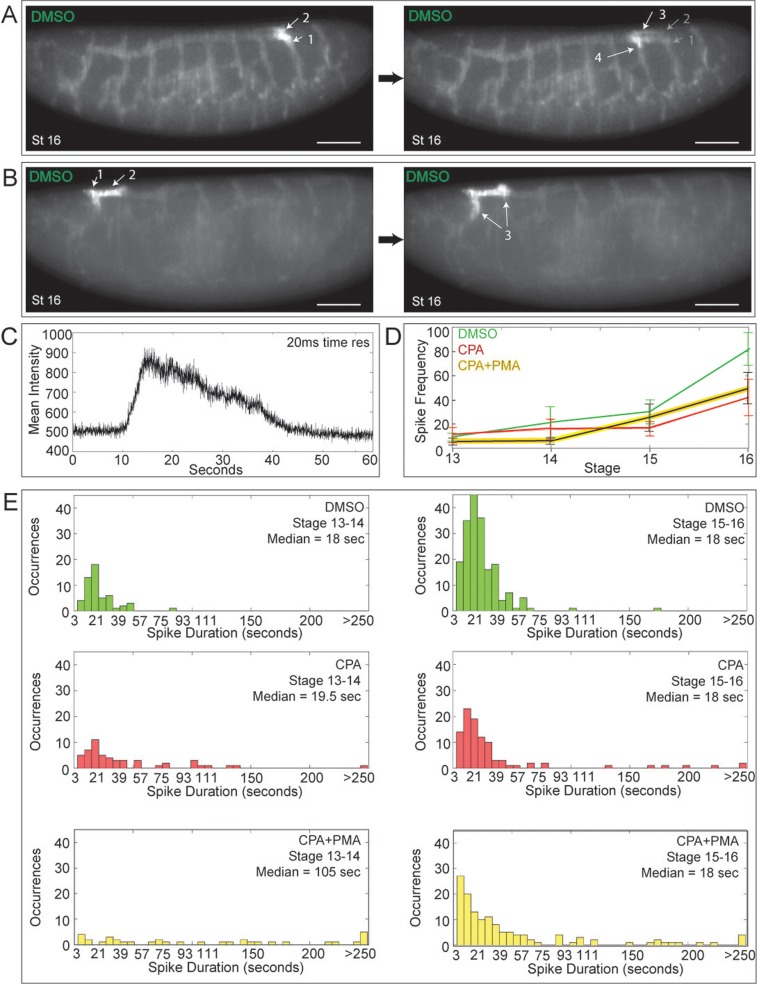
**Live Ca^2+^ imaging shows that impulses propagate the *Drosophila* tracheal network.** *Btl:GCamp3* embryos were imaged in 3D+time (3 sec time resolution) from stage 13 to 16 using two-photon light-sheet microscopy. (A,B) Time-lapse imaging of two control embryos at stage 16 reveal Ca^2+^ pulses propagating through electrically coupled cells once the tracheal network has fused, such as between adjacent transverse connectives via the dorsal trunk (A), or bidirectionally (B) (follow arrows in numbered sequence). The time points shown are (A) 21 and (B) 9 sec apart. Scale bars: 50 µm. (C) At 20 ms time resolution, a typical Ca^2+^ spike shows a fast upstroke and slower decay. (D) The mean Ca^2+^ spiking frequency and SEM for control, CPA-treated, and CPA+PMA-treated embryos at each stage are plotted (*n*>3; mean±s.e.m.). Spike frequency increases with embryo age. Compared to controls, the frequency of Ca^2+^ impulses at later stages is diminished by SERCA blockade, even in the presence of the PKC activator PMA. (E) Histograms of Ca^2+^ spike duration at stages 13-14 (left) and 15-16 (right) for embryos treated with DMSO (green), CPA (red) and CPA+PMA (yellow). In contrast to DMSO controls, embryos treated with CPA±PMA feature two types of Ca^2+^ pulse: (1) normal duration (clustered around 18 sec) and (2) prolonged with slow decay (see Movie 7).

Live Ca^2+^ imaging of *Drosophila* tracheogenesis revealed dramatic propagating impulses of tracheal cell Ca^2+^ (Fig. 4A,B; Movies 6 and 8). These often occurred amongst leader cells as the tracheal tubules fused (Fig. 3C black arrows). A typical impulse comprised a fast increase in cytoplasmic Ca^2+^, followed by a slower decay (Fig. 4C). The frequency of Ca^2+^ impulses increased with embryo stage (Fig. 4D), with most lasting around 18 sec (median) (Fig. 4E). A few were longer, propagating back and forth between neighboring cells. SERCA-inhibited embryos retained some impulses of normal duration, likely identifying cells with low levels of inhibitor, but featured a second population of more prolonged Ca^2+^ elevations with much slower signal decay (Fig. 4E; Movie 7). CPA significantly reduced the frequency of Ca^2+^ impulses compared to controls by stage 16 (Fig. 4D). PMA rescue of budding in CPA-treated embryos did not restore impulse frequency or duration (Fig. 4D,E), nor did it restore the presence of Ca^2+^ impulses commonly seen when leader cells meet and fuse with their neighbors (Fig. 3G). Alterations in these impulses, therefore, do not explain the branching defects observed. Thus, the live Ca^2+^ imaging shows that branching persists (or resumes) only when the leader-trailer Ca^2+^ differential is maintained (or restored). This pinpoints the Ca^2+^ differential as a key element in setting up the *directed* migration required for budding in this fundamental model of epithelial branching morphogenesis.

### SERCA controls non-endodermal budding, again by regulating cell migration

Parallel studies of other systems corroborate the findings in the tracheal branching. Stereotyped neural branching in *Drosophila* (Klämbt et al., 1991) was disrupted by SERCA inhibition with CPA. The parallel longitudinal tracts of the wild-type nerve cord (Fig. S7A) become disordered, with discontinuities and aberrant midline crossing following SERCA inhibition (Fig. S7B). Peripheral nerves are also disorganized and sometimes absent. Washout of CPA mostly corrects the structure of the central nerve cord, except that the longitudinal tracts are slightly more widely spaced than in controls (Fig. S7C). Neural branching was corrected by activation of PKC by co-treatment with PMA (Fig. S7D). Furthermore, the aberrations resulting from SERCA inhibition with CPA again support a cell-autonomous role for SERCA activity (Fig. S7E). Within individual embryos, some portions of the nerve cord and peripheral nerve projections are disrupted (Fig. S7E, red arrowheads), while adjacent segments can generally be normal (white arrowheads).

**Fig. 5.**
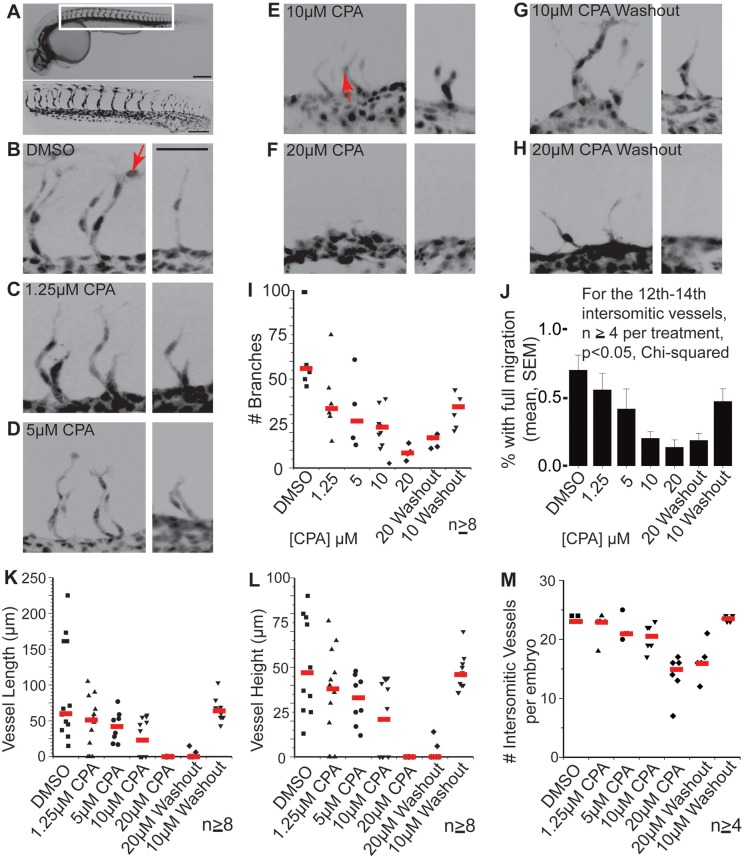
**SERCA function controls the rates of mesodermal migration and budding.** (A) Wild-type 28 hpf Tg(kdrl:eGFP) zebrafish embryo demonstrates intersomitic vasculature seen by wide field (top, scale bar: 200 μm) and higher magnification below (scale bar: 100 μm). For A-H, images are inverted for improved clarity. (B-H) Paired confocal images show 3D reconstructions of the 14th-15th and 20th intersomitic vessels from embryos treated with (B) DMSO, (C) 1.25 µM CPA, (D) 5 µM CPA, (E) 10 µM CPA, (F) 20 µM CPA (G) 10 µM CPA washed out after 2 h, and (H) 20 µM CPA washed out after 2 h. Varying CPA dose results in dose-dependent reduction in vascular budding until at 20 μM, budding is reversibly suspended. Budding resumes after CPA washout. Scale bar: 50 µm for all images. Red arrows indicate distal-most positioning of nuclei in B and E (see caption for I,J). (I,J) Plots show, for each treatment, the collective number of branches (red lines are medians) on vessels 13-16 which form in the middle of the treatment (I) and the proportion of branches with cell nuclei at tip positions (J): compare the positions of nuclei (arrowed) in B versus E. Error bars in J indicate the s.e.m. In a parallel dose-dependent manner, CPA reduces branch numbers (I) and distal migration of endothelial cells (J) with resumption of branching and migration upon CPA washout. (K-M) Vessel path length (K), linear vessel height (L), and the total number of intersomitic vessels per embryo (M) measured in 3D show similar CPA dose- and time-dependent reductions in branching. Data in K and L are shown for the 20th intersomitic vessel, which forms during the treatment (red lines are medians).

In zebrafish, both the initiation and elongation of intersegmental vasculature branches (Childs et al., 2002) was reduced by CPA (Fig. 5A-F). Incubating the zebrafish embryos in escalating concentrations of CPA (1.25, 5, 10, 20 μM) resulted in dose-dependent reductions in vessel number, size, and branches (Fig. 5I,K-M). CPA treatment reduced the fraction of vessels with nuclei present at the distal tips, suggesting that endothelial cell migration was impaired (Fig. 5J; compare arrowed nuclei in Fig. 5B and E). After CPA washout, endothelial budding resumed, with increasing branch numbers and sizes, as well as proportionately more branches featuring cell nuclei in distal positions (Fig. 5G-M). Thus, titrating the level of SERCA function with CPA dose tightly controls the extent of both endothelial cell migration and bud iteration in concordance with the level of SERCA activity. Together, these findings show that SERCA regulates budding in tissues from all three germ layers: ectoderm, mesoderm, and endoderm.

### SERCA regulates cell migration to control the onset and rate of bud iteration in mammals

**Fig. 6.**
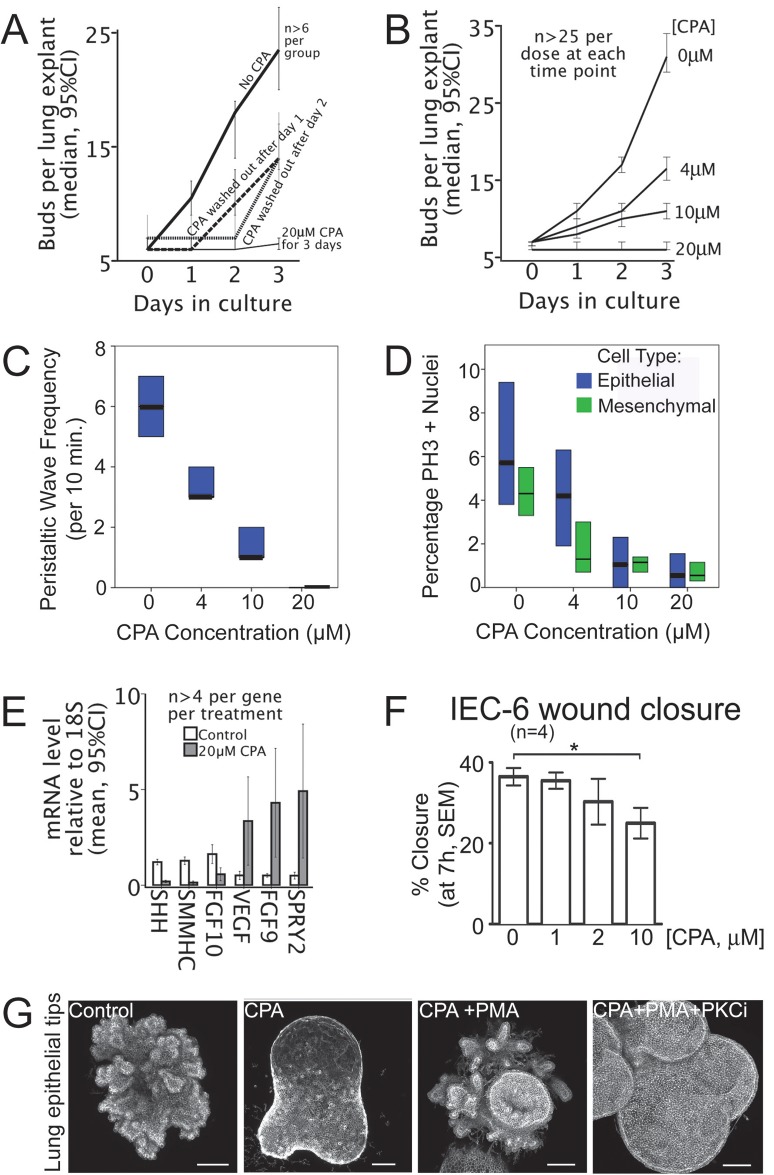
**SERCA function controls the onset and rate of lung branching.** (A) SERCA function dictates the onset of new buds*.* Plot of lung bud count versus time in culture for E13 rat lung explants shows the budding rate in controls (no CPA), lack of budding with 20 μM CPA, and resumption of budding when CPA is removed. (B) SERCA function titrates budding rate*.* Bud count plotted against days in culture. The normal accretion of buds is shown in the absence of CPA (0 μM). Escalating the CPA dose controls the budding rate. At 20 μM, branching is arrested. (C) The frequency of airway peristaltic waves decreases with escalating CPA dose, with statistical significance between each treatment group (*P*<0.05, Mann-Whitney U test). Median and interquartile range (IQR) are plotted, *n*>10 for each treatment. (D) Proliferation of lung epithelial and mesenchymal cells decreases with escalating CPA dose, with statistical significance between treatments for each cell type, except 10 µM and 20 µM are equivalent (*P*<0.05, Mann-Whitney U test). Median and IQR of PH3 positive nuclei are plotted, *n*>24 for each treatment. (E) SERCA blockade is associated with downregulation of lung morphogens SHH, FGF10, and SMMHC (smooth muscle myosin heavy chain), and significant upregulation of SPRY2, FGF9 and VEGF (qRT-PCR). Error bars indicate 95% confidence interval. (F) SERCA inhibition impairs epithelial cell migration. Plot of percentage closure at 7 h (mean±s.e.m.) of a standardized wound in a confluent monolayer of IEC-6 intestinal epithelial cells treated with 0, 1, 2, or 10 μM CPA. Wound closure is significantly reduced by 10 μM CPA (*P<0.05, one-way ANOVA and Bonferroni multiple comparisons test). (G) Epithelial SERCA blockade halts budding and is rescued by PKC activation. Epithelial tips isolated from E12.5 murine lungs bud in Matrigel with FGF10. Control epithelial tips bud extensively (left panel). 10 μM CPA abolishes budding, despite co-incubation with FGF10 (2nd panel). Budding is rescued by co-treatment with PKC activator (100 nM PMA) (3rd panel). Budding is re-inhibited by PKC inhibition (2.22 μM *Bisindolylmaleimide I* Hydrochloride), demonstrating that PMA rescue is mediated by PKC (right panel). Scale bars: 100 μm.

In both rat and mouse embryo lung explants, 20 μM CPA completely suspended branching for the duration of the 3-day treatment. Following washout of CPA after either 1 or 2 days, branching resumed, and the next scheduled branch emerged (Fig. 6A). Lower doses of CPA (4, 10 μM) showed dose-dependent effects on lung explant branching rate (Fig. 6B). Parallel dose-dependent changes were observed in the frequency of airway Ca^2+^ waves (Fig. 6C) and rates of cell proliferation (Fig. 6D). SERCA inhibition altered the levels of intrinsic lung morphogens: SHH, FGF10, FGF9 and mSpry2 (Fig. 6E). Thus, SERCA activity controls the onset and rate of lung budding in mammals and affects proliferation and the expression of pulmonary morphogens.

SERCA blockade perturbed epithelial migration assayed in culture. Migration closes standardized wounds in a mammalian epithelial monolayer (IEC6 cells) without reliance on cell proliferation or branching. SERCA blockade with CPA slowed wound closure in a dose-dependent manner (Fig. 6F) and abolished EGF-stimulated migration (relative migration for controls+EGF: 135±21.6 versus EGF+CPA: 100±10; *P*=0.04; *n*=3).

In mammalian airway epithelium, loss of epithelial SERCA function inhibited budding. Epithelial tip explants from embryonic mouse lung, isolated in culture, bud independently of regulators of budding in the surrounding mesenchyme, such as nerves and vessels (Jesudason et al., 2005; Englund et al., 1999; Del Moral et al., 2006; Bower et al., 2014). SERCA blockade reduced or suspended budding of these isolates (Fig. 6G). During SERCA blockade, FGF10 was unable to rescue budding, but PKC activation using PMA did rescue budding (Fig. 6G). The PKC inhibitor, *Bisindolylmaleimide I Hydrochloride* abolished this PKC-mediated rescue (Fig. 6G); PKC inhibition alone did not reprise the CPA phenotype (Fig. S8A). SERCA inhibition halted branching independently of Ca^2+^-dependent mechanotransduction (Malek and Izumo, 1996; Mammoto and Ingber, 2010; Frey et al., 2004): inhibitors of ROCK, PKC, PLC and Rac1 neither reproduced nor alleviated the CPA phenotype (Fig. S8). Thus, PKC-regulated SERCA is specifically required in the mammalian airway epithelium for budding.

### SERCA controls budding without inducing major changes in cell shape

Comparison of cell geometries between wild-type and SERCA-inhibited mouse airway epithelium revealed similar linear relationships between ‘area’ and ‘perimeter’ and no differences in cell sidedness (Fig. S9A,B). Likewise, there was no change in cell shape between wild-type and *serca* RNAi cells in the *Drosophila* air sac (Fig. S9C,D). Together, these data show that SERCA provides conserved control of budding through Ca^2+^-directed cell migration, rather than by regulating proliferation or cell shape.

## DISCUSSION

SERCA performs diverse regulatory functions, ranging from roles in periodic contractility in muscle to ER stress and protein folding (Sanyal et al., 2006; Caspersen et al., 2000). Our findings reveal a new function for SERCA, as a conserved controller of iterative budding. The initiation of new buds and encoding of the timing of formation of these buds has been proposed to be controlled by growth factor morphogens (Metzger et al., 2008; Morrisey and Hogan, 2010). Specifically, FGF10 acting on airway epithelial FGFR2b (in mammals) or Branchless acting on Breathless (in *Drosophila*) are required for proper branching of mammalian lungs or *Drosophila* trachea, respectively (Glazer and Shilo, 1991; Klämbt et al., 1992; Min et al., 1998). Unidentified morphogens have also been proposed to act as a ‘branching clock’ that work with FGF signaling to coordinate the branching program (Metzger et al., 2008). In contrast to this hypothesis that unidentified growth factor morphogens serve as the ‘clock’ to direct the timing of branching, we show here that SERCA is a central organizer that directs the onset and rate of budding. Morphogens must operate upstream of SERCA, because SERCA blockade stalls the branching program, while supply of exogenous morphogens (e.g. FGFs) is insufficient to overcome this blockade. Thus, we propose that SERCA must integrate inputs from morphogens like FGF and establishes a differential in Ca^2+^ levels at branching tips to indicate the timing for directed cell migration and branch formation.

This novel role of SERCA as a central organizer of branching seems highly conserved, as branching in both invertebrates and vertebrates, as well as tissues from all germ layers, requires SERCA. In all these systems, branch iteration rate is determined by the level of SERCA function; these effects are mediated by controlling cell migration. SERCA's effects are not mediated by altering cell shape, and do not require alterations in proliferation. Our live Ca^2+^ imaging in *Drosophila* reveals that SERCA directs cell migration at branch points by establishing a local Ca^2+^ differential, where the Ca^2+^ level is higher in the leading cell that migrates to form a new branch. The cells trailing behind it maintain comparatively lower Ca^2+^ levels. Loss of this local Ca^2+^ differential halts migration and branching. Reinstatement of this local Ca^2+^ differential, whether by lifting of SERCA blockade or by PKC activation, restores cell migration and branching.

Beyond the Ca^2+^ differential revealed by our light-sheet imaging of *Drosophila* embryos, episodic Ca^2+^ impulses were observed to propagate through the tracheal epithelium as the cells migrate and fuse to form their branched network. These propagating Ca^2+^ waves have been predicted by computational modeling (Kang and Othmer, 2007), yet they do not appear to be important for branch iteration, raising the question as to their function. A recent publication on tracheal tube anastomosis did not implicate these whole-cell Ca^2+^ impulses in membrane fusion (Caviglia et al., 2016). The increase in frequency of these impulses upon fusion of cells from adjacent segments suggests they may be a response to cell-cell contact, which could in turn modulate cell membrane machinery. Similar Ca^2+^ impulses have been described in other cell types, such as in fungi following contact with a pathogen (Kim et al., 2012). The remarkable similarity of these Ca^2+^ impulses from animals to fungi suggests that they are highly conserved and may have been adapted by evolution to suit each specific cellular environment. The function of these impulses may, alternatively, relate to maintenance or elongation of the branched network that has formed. Indeed, in mammalian lung, periodic Ca^2+^ waves course through airway smooth muscle, inducing waves of contractility. These waves are thought to mechano-regulate branching morphogenesis, whereby abolishing the Ca^2+^ waves impairs airway growth and elongation (Jesudason et al., 2005).

Our results consistently demonstrate that SERCA instructs budding across germ layers, tissue types, and species, suggesting that the role of SERCA may be more broadly generalizable. A conserved regulator simplifies our understanding of how a vast array of branched tissues could arise from one platform, and specialize based on local morphogen inputs. Thus, our findings may unite disparate observations of Ca^2+^ signaling involvement in other types of branching, such as axonal pathfinding, plant gravitotropism, angiogenesis, and endothelial wound healing (Usachev et al., 2006; Urbina et al., 2006; Evangelista et al., 2012). A centralized control of branching also holds implications for understanding a range of disease mechanisms. Regarding the lung, the significance of reduced epithelial SERCA function has been highlighted in human and animal studies of asthma (Cantero-Recasens et al., 2010; Mahn et al., 2009) as well as in other burgeoning diseases such as cystic fibrosis (Ahmad et al., 2009), lung fibrosis (Lawson et al., 2011) and lung cancer (Korosec et al., 2006). Our study suggests that these oft intractable pulmonary challenges may feature SERCA-related lesions of cell migration. Examples include airway remodeling in asthma, alveolar remodeling in fibrosis, or lung cancer invasiveness. More generally, altered SERCA expression or function has been associated with numerous cancers (Arbabian et al., 2011; Papp and Brouland, 2011; Papp et al., 2012), and changes in SERCA expression have been reported during cell lineage differentiation (Flores-Peredo et al., 2017; Lacabaratz-Porret et al., 2000; Launay et al., 1999). Therefore, a wider opportunity may lie in determining how SERCA-mediated Ca^2+^ switching helps cells find not just their route, but also their fate.

## MATERIALS AND METHODS

### Ethics statement

Protocols complied with NIH Guide for the Care and Use of Laboratory Animals. Mouse and rat protocols were approved by the Institutional Animal Care and Use Committee at Children's Hospital Los Angeles (IACUC protocol #252) or with UK Home Office License (Animal Scientific Procedures Act 1986). The zebrafish protocol was approved by the Caltech Institutional Animal Care and Use Committee (1227-09).

### RNA extraction

*Drosophila* embryos and lung explants were snap frozen. RNA was extracted using RNeasy Mini Kit according to Qiagen's handbook. The concentration of RNA was determined at 260 nm using a NanoDrop ND-1000 spectrophotometer. The A260/A280 ratio was assessed for RNA purity.

### Quantitative RT-PCR

First-strand cDNA synthesis was initiated from 0.2 µg total RNA and performed using M-MLV reverse transcriptase from Promega, Madison, WI, USA. The cDNA was diluted to 100 µl with nuclease-free H_2_O and stored at –30°C. For qRT-PCR, 5 µl cDNA was used to analyze transcript targets using SYBR Green QPCR Master Mix (Agilent) and specific primer sets. Primers used for mouse were: *Fgf9* (F: CGGCACCAGAAATTTACACA; R: CGGCACCAGAAATTTACACA), *Fgf10* (F: CACATTGTGCCTCAGCCTTTCC; R: CCTGCCATTGTGCTGCCAGTTAA), *Shh* (F: GGAAAACACTGGAGCAGACC; R: CCACGGAGTTCTCTGCTTTC), *Sm-mhc* (F: AGGAAACACCAAGGTCAAGC; R: CCCTGACATGGTGTCCAATC), *Spry2* (F: TTGTGGTTTGCAGTGAGAGG; R: TCTTCGCCTAGGAGTGTTGG) and *Vegf* (F: ATGTGACAAGCCAAGGCGGTG; R: TGGCGATTTAGCAGCCAGATA). The MX3000P^®^ Multiplex Quantitative QPCR System (Agilent) was used for all reactions and MxPro software for analysis. Transcripts were quantified using the relative standard curve method. Real-time qRT-PCR efficiency was determined by analysis of serial dilutions of a pool of cDNA sample. All reactions were run in duplicate or triplicate; mRNA expression per gene was normalized to 18S (F: TCCGATAACGAACGAGACTC; R: CAGGGACTTAATCAACGCAA).

### *Drosophila* stocks and crosses

Stocks were obtained from the Bloomington *Drosophila* Stock Center (Indiana University, USA), the Vienna *Drosophila* RNAi Center (Institute of Molecular Biotechnology and Research Institute of Molecular Pathology, Vienna, Austria) and kindly provided by Dirk Bohmann's laboratory (University of Rochester, USA). Fly stocks were maintained at 25°C with a 12 h light:12 h dark photo cycle, on *Drosophila* yeast/glucose medium. Spatially restricted gene silencing of *serca* in the air sac was achieved using the *Drosophila GAL4-UAS* system and RNAi constructs (Brand and Perrimon, 1993; Dietzl et al., 2007): w*; btl-GAL4, UAS-Act5C. GFP was crossed to the RNAi line *w^1118^; P{GD436}v4474* in order to specifically knockdown *serca* and express *gfp* in tracheal cells: *w^1118^* was crossed as a control. For more potent *serca* knockdown, a similar experiment was carried out, but using RNAi recombined with ectopic *dicer2* (*41*) on the second chromosome (*w^1118^; P{GD436}v4474, UAS-dicer*) or two different RNAi constructs recombined (*w^1118^; P{GD436}v4474, P{KK107371}v107446*): in these cases it was necessary to temporally repress the knockdown until the larval stage, using a temperature sensitive GAL80 line (w*; btl-GAL4, UAS-Act5C:GFP; tubGAL80ts), to circumvent early lethality. A similar experiment was carried out using *btl-GAL4, UAS-GFP, tubGAL80ts, esg-LacZ* to assess *escargot* expression.

*hsFlp; act>y>GAL4, UAS-GFP; btl-mRFPmoe* was similarly crossed to *w^1118^; P{GD436}v4474* or *w^1118^* to create GFP-labeled *serca* loss-of-function or control flip-out clones. In this experiment the tracheal system was labeled with RFP.

### Collection of *Drosophila* embryos and larvae

Embryos were collected on apple juice agar plates smeared with yeast paste. A pre-lay collection helped synchronize subsequent collections which were carried out over 1-4 h. Embryos were aged on agar plates at 25°C to reach the desired stage. They were removed by washing with distilled water through a fine mesh sieve (Sefar Nitex 120 µM mesh, Sefar Ltd., Bury, UK). The chorion was removed by placing the sieve into thin household bleach for 1-2 min. The bleach was drained off and embryos were washed with distilled water. Embryos were removed with a damp paintbrush and dissociated for RNA extraction or Ca^2+^ imaging. For larval collection, embryos were collected over 4–8 h and allowed to develop to the required stage at 25°C.

### Immunohistochemistry of *Drosophila* imaginal discs

Fixation and staining of *Drosophila* imaginal discs was performed in ‘watch-glass’ containers. Wing discs were fixed in 4% paraformaldehyde (PFA) for 20 min, then washed twice in PBS for 20 min. A blocking step was carried out for 2 h at 25°C using 5% fetal calf serum in PBST (PBS+0.1% Triton X-100). Discs were incubated with Anti-β-Galactosidase (1:1000, Promega, Madison, WI, USA) and Anti-phospho-Histone H3 Ser10 (1:500, Cell Signaling Technologies, Danvers, MA, USA) primary antibodies overnight at 4°C (Hendzel et al., 1997; Wang et al., 2010). Four 20-min washes were performed with PBST before incubation with secondary antibody. Alexa Fluor® (1:500, Invitrogen) secondary antibody was incubated for 2 h at 25°C. We washed four times 20 min washes in PBST and then a final wash of 20 min in PBS to remove detergent. Wing discs were mounted on microscopy slides with VECTASHIELD® Mounting Medium (Vector Laboratories). Slides were kept dark at 4°C to reduce fluorophore fading.

### Fluorescent Thapsigargin staining of *Drosophila* embryos

Embryos were fixed in heptane and 5% PFA in PBS for 15 min at room temperature. The PFA was removed and 100% methanol added, and the embryos were shaken to remove the vitelline membrane. The heptane was aspirated and embryos were rehydrated to PBS, then blocked for two times 30 min in PBS+0.05% TritonX-100+0.5% BSA. Embryos were incubated in 5 µM red-fluorescent BODIPY® TR-X thapsigargin (Invitrogen) for 2 h at 25°C, then washed in PBST for two times 30 sec two times 5 times min, and two times 30 min. Embryos were mounted on microscopy slides with VECTASHIELD® Mounting Medium (Vector Laboratories, Peterborough, UK) using coverslip spacers and sealed with clear nail polish.

### Generation of flip-out and mitotic clones

*serca* loss-of-function ‘flip-out’ clones were produced using the heat-shock-induced FLP-recombinase system (Duffy et al., 1998; Struhl and Basler, 1993). For clonal analysis, embryos were kept at 25°C until late L2 larval stage. Vials containing larvae were then placed in a 37°C water bath for between 15 min and 1 h to activate the flippase (FLP) recombinase which is under the control of a heat-shock promoter (*hs*). The larvae were returned to 25°C until the late L3 when they were dissected. The presence or absence of clones within the discs was determined by fluorescence microscopy.

### Larval micro-dissection

Larvae were washed and then dissected in ice cold PBS. Larvae were transected at the abdomen and inverted over forceps. The wing disc was dissected from the thoracic trachea and transferred to cold PBS.

### Imaging Ca^2+^ levels in dissociated *Drosophila* embryos

Embryos were collected and dechorionated as above. 100-200 embryos were placed in a micro-centrifuge tube containing 800 µl of GIBCO™ Schneider's *Drosophila* medium (Invitrogen) and dissociated using a sterile pestle. The suspension was centrifuged at 40 ***g*** for 5 min and the process repeated. The cell suspension was diluted to 1200 µl with medium. 300 µl of this was loaded into each chamber of a 4-well Lab-Tek™ II Chambered Coverglass (Nunc, Thermo Fisher Scientific, Rochester, NY, USA) pre-coated with Poly-L-Lysine (Sigma-Aldrich) for 1 h and washed with sterile water. Cells were loaded using 1 µM Fluo-4 (Invitrogen) for 1 h. Confocal microscopy was performed as described below. When required, thapsigargin (Sigma-Aldrich) was added to give a concentration of 2 µM. For real-time Ca^2+^ release experiments, cells were imaged with a 20× objective on a Zeiss LSM 710 (Carl Zeiss Ltd, Hertfordshire, UK) microscope using maximum scan speed without averaging.

### Air sac microscopy and image processing

We imaged the dorsal air sac primordium (ASP) from third instar larvae (Sato and Kornberg, 2002). Slides were imaged using a Zeiss LSM 710 (Carl Zeiss Ltd) laser scanning confocal system with an inverted Axio Observer.Z1 microscope. Zeiss Fluor 20×/0.75 air and 40×/1.3 oil immersion objectives were used. GFP fusion proteins were excited at 488 nm, using an Argon laser and detected maximally at 509 nm. mRFP fusion proteins and Alexa Fluor 555 were excited at 543 nm using a He/Ne laser and detected maximally at 607 and 565 nm, respectively. The pinhole was set at ∼1 AU. When z-stacks were taken, we used the slice thickness specified by the software for 1 AU (usually 0.5–2 µm). Images were captured using Zen 2010 (Zeiss) software, exported in tagged image file format (TIFF) and edited in Adobe Photoshop CS5 (Adobe Systems Europe Ltd, Maidenhead, UK). When z-stacks were produced, images are presented as single slices, unless a projection is specified. When a 3-dimensional (3D) projection was required, stacks were rendered using Zen 2010 software (Zeiss). If image brightness was altered for publication, this change was standardized across groups to retain comparability.

### Embryo preparation for *Drosophila* embryonic tracheal and nerve studies

*Drosophila* embryos were collected for 3 h from wild-type or *w; Btl:Gal4, UAS-dsRed-nuclear localization signal, UAS-actinGFP* flies. Embryos were dechorionated in 50% bleach for 2 min and rinsed in tap water. Embryos were transferred to nylon cell strainers (BD Falcon 2360) and permeablized with 90% D-limonene, 5% cocamide DEA, 5% ethoxylated alcohol (Rand et al., 2010; Strecker et al., 1994). This embryo permeablization solvent (EPS) was diluted 1:10 into modified basic incubation medium (MBIM) for treatment of the embryos as described, except that the malic acid was not added (Rand et al., 2010). Embryos were permeablized for 30 sec, washed four times in PBS and twice in PBST and distributed onto nylon cell strainers or Whatman paper for incubation. Embryos were incubated on cell strainers or Whatman paper in 6-well dishes in contact with drug (20 µM CPA, 100 nM PMA) or DMSO diluted in MBIM until they reached stages 15-16 by gut morphology. For washout experiments, the drug solution was removed at stage 12 and the embryos were rinsed and then incubated for the duration of the time in MBIM without drug.

For whole-mount preparation, when embryos reached stages 15-16, they were transferred to glass vials and treated with heptane and 5% PFA in PBS for 15 min at room temperature to fix. The PFA was removed and 100% methanol added, and the embryos were shaken to remove the vitelline membrane. The heptane was aspirated and embryos were rehydrated to PBS and transferred to PBT (PBS+0.05% TritonX-100+0.1% BSA).

For antibody staining, embryos were blocked for 1 h at room temperature in 5% normal goat serum (NGS) then incubated with primary antibodies 2A12 (1:2) (Developmental Studies Hybridoma Bank), rabbit anti-GFP (1:1000) (Abcam AB290) and 1D4 (1:3) (Developmental Studies Hybridoma Bank) in PBT+2% NGS overnight at 4°C. Embryos were washed six times for 30 min at room temperature in PBT then blocked for 20 min in 5% NGS. Goat anti-mouse IgG1, goat anti-mouse IgM, goat anti-rabbit secondary antibodies (Invitrogen) were used at 1:500 in PBT+2% NGS overnight at 4°C. Embryos were washed six times for 30 min at room temperature and transferred to PBT/14% glycerol then mounted on slides.

Fillet preparations were performed as described (Lee et al., 2009). Confocal and two-photon tiled z-stacks were collected with a Zeiss LSM 510 meta microscope with a 2 µm pinhole (for confocal) and 1.5 µm z-step interval. Images were assembled using Fiji stitching plugins (Preibisch et al., 2009) and viewed in 3D using Imaris software (Bitplane).

Severity of tracheal phenotypes for each treatment group was scored as follows: a ‘severe’ tracheal phenotype was defined by the presence of breaks, missing sections, grossly abnormal structure, and in the case of the washout samples, the complete formation of a supernumerary ‘lateral’ trunk. A ‘moderate’ phenotype was classified as those with slightly abnormal structure, excessively sprouty and tortuous branches, and for the washout samples a partially formed extra ‘lateral’ branch. ‘Normal’ was defined as having generally classic structure.

### Dynamic imaging of live *Drosophila* embryos and tracking of lateral trunk cell migration

*Drosophila* embryos from the transgenic line *w; Btl:Gal4, UAS-dsRed-NLS, UAS-actinGFP* were collected and permeablized as described above. Embryos were treated with DMSO (control) or 20 µM CPA and screened for fluorescence around stage 12-13. Fluorescent embryos were mounted lateral side down on glass coverslips with heptane glue, or in coverslip-bottomed dishes in 1% agarose molds. The latter were covered with 1% low-melting point agarose and incubation medium and imaged through the coverslip with water immersion fluid. We used the LD-C-Apochromat 40×/1.1W Korr UV-VIS-IR objective on a Zeiss LSM 510 meta or Zeiss LSM 5 Exciter confocal microscope with 488 nm and either 543 or 561 nm lasers. Z-stacks of 318×318×64 µm were collected with 0.62×0.62×2 µm^3^ voxel size, every 3 to 3.5 min.

Datasets were compiled and registered using Imaris 7.6 (Bitplane). Individual cells destined to migrate into the lateral tracheal trunk were manually tracked from stage 14 to 16. Positions of the cells over the time course were exported to Matlab. For pairs of tracheal cells in adjacent segments, the direction of travel of one cell relative to the other was calculated, and a vector was plotted for each pair to compare the convergence or divergence of all pairs from each treatment together. The separation between pairs of cells that should migrate together to form the trunk was similarly measured to determine their convergence. Movies were made using Imaris 8.4 (Bitplane), ImageJ (NIH), and FFmpeg (www.ffmpeg.org).

### Light-sheet imaging of Ca^2+^ dynamics in live *Drosophila* embryos, data processing and analysis

*w;Btl:Gal4, UAS-GCamP3* embryos expressing the GCamP3 Ca^2+^ indicator in tracheal cells were permeablized and treated with DMSO (control) or 20 μM CPA (mutant) or 20 μM CPA + 100 nM PMA (rescue) as described previously. When the embryos reached approximately stage 13 by gut development, they were aligned in a row on agarose, lateral side down, and gently touched to a heptane glue-coated glass cylinder to mount them to the cylinder with one lateral side exposed. The glass cylinder was quickly mounted into the sealed, fluid-filled sample chamber of the two-photon light-sheet microscope (Truong et al., 2011). Light-sheet microscopy illuminates a single z-slice at a time, minimizing phototoxicity, which is further reduced by using infrared excitation. The image of the entire x-y plane in focus can be captured simultaneously with a camera since there is no out-of-focus excitation. This affords extremely high time resolution. The bi-directionally scanning light-sheet on this microscope also ensures even illumination at each end of the x-y plane. The span of the axial (z) imaging depth was set to capture the entire tracheal network on the side of the embryo facing the collection objective.

Embryos were imaged from approximately stage 13 to stage 16 using 940 nm illumination at the same laser power and exposure time and with the same camera detection gain across all samples. The z-slice thickness was 1.5 µm. The x-y resolution of the image was 0.8 µm/px. An entire z-stack capturing the tracheal network on one side of the embryo was collected in less than 3 sec permitting 3 sec time resolution between time points with resting time for the embryo between each scan. Embryos showed no sign of phototoxicity, and several hatched during the course of the imaging. For embryos that had been treated with CPA or CPA+PMA, the same concentration of drug was added to the water in the sample chamber to maintain the drug treatment while imaging.

Autofluorescence from the embryo surface resulted in a ‘shell’ when images were reconstructed in 3D. To remove this, a Matlab script was developed using the Canny edge detection algorithm to identify all sharp signal transitions. The ‘shell’ was identified by the first and last columns with data in each row. For each individual image, a mask was uniquely created to eliminate the ‘shell’, employing an R loess smoothing function to obtain a smooth boundary on the inside of the ‘shell’.

Masked images were reviewed using ImageJ to find the z-slices with lateral trunk cells. Matlab was used to generate a summative projection of each time point z-stack that included the lateral trunk but minimized contributions from gut autofluorescence. Other projections were also made to include more of the tracheal network, albeit with more gut as well. The summed images of the lateral trunk were imported into ImageJ for analysis.

In the summed z-stack, the total fluorescence intensity of a lateral trunk cell was represented in the 2D projection for that time point. A region of interest, always with the same area, was drawn within the cell of interest. Individual lateral trunk cells were manually tracked across each time point of the datasets during the course of lateral trunk formation. A macro modified from that used by Kim et al. (2012) was used to record the sum total intensity value within the region of interest at each time point (Kim et al., 2012). This process was repeated for each cell tracked in each of the datasets for the embryos treated with DMSO, CPA, and CPA+PMA. For homozygote embryos (such as in Fig. 3C), the intensity values were halved to normalize to heterozygote embryos.

The frequency of Ca^2+^ spikes was determined by counting impulses after summing to include the complete tracheal network on the half of the embryo that was imaged. Spike duration was likewise determined by counting the number of time points across which an individual impulse lasted. The error in pulse duration is ±3 sec. Data were plotted with Matlab.

### Zebrafish intersomitic vessel studies

Transgenic zebrafish with the VEGF receptor promoter driving eGFP expression [Tg(kdrl:eGFP)] express cytoplasmic eGFP in endothelial cells. These fish were crossed with wild-type fish and embryos collected and incubated until 21 h post-fertilization (hpf). eGFP-positive embryos were sorted and dechorionated. Embryos were incubated with cyclopiazonic acid (CPA) or DMSO from 22 hpf until 28 hpf in 1.5 ml of egg water. For the washout study, embryos were incubated in 10 µM or 20 µM CPA for 2 h, rinsed three times in egg water and incubated for 4 h without drug. After 6 h of incubation, embryos were rinsed three times in egg water and fixed overnight at 4°C in 4% PFA. Embryos were mounted in agarose molds and imaged with a Zeiss LSM 510 meta microscope. Z-stacks were collected using: 488 nm laser excitation, 2 µm pinhole, 2 µm z-step interval. Z-stacks were assembled, and Imaris software (Bitplane) was used to generate 3D images and measure vessel dimensions in 3D.

### Lung cultures

Embryos from mice or rats were harvested on day 11.5 or 13 of gestation, respectively (vaginal plug positive=day 0). Lungs were dissected and cultured as described (Jesudason et al., 2000). Cyclopiazonic acid (CPA) (Sigma-Aldrich Company Ltd., Dorset, UK or equivalent) was filter sterilized and added for final concentrations of 2–20 µM. Lung morphometry was assessed with terminal bud count. Peristaltic wave frequency was measured in 10 min periods (Jesudason et al., 2005). At the end, lung cultures were homogenized for RNA extraction or prepared for histology. Mitotic cells were labeled with Anti-phospho-Histone H3 Ser10 staining (Brand and Perrimon, 1993). Epithelial tip cultures were performed as described, but without enzymatic digestion (Bellusci et al., 1997). Mechanotransduction inhibitors used included: PKCi (Bisindolylmaleimide I Hydrochloride, #203290 Calbiochem), PLCi (L108 Edelfosin, #BML-L108, Enzo Life Science), ROCKi (Y26732, #Y0503, Sigma), RACi (NSC 23766, #553502, Millipore).

### Epithelial migration assay

Confluent IEC-6 intestinal epithelial cell (ATCC CRL-1592) monolayers were treated with 0, 1, 2, or 10 μM CPA after wounding with a rotating silicone tip (Frey et al., 2004). The latter CPA dose was tested ±10 ng/ml EGF. Wound closure rates were determined by time-lapse microscopy.

### Cell shape analyses

Lung epithelial tips were fixed in 4% formaldehyde (w/v) solution and stored at −20°C in 70% ethanol. F-actin fluorescence staining was performed by permeabilizing with 0.5% Triton X-100 in PBS for 10 min at room temperature, and then staining with Rhodamine phalloidin (Molecular Probes R415, 5 units/ml) and DAPI (10 ng/µl) in PBS containing 1% BSA overnight at 4°C. Confocal z-stacks were acquired with an LSM 700 confocal system mounted on an AxioObserver. Z1 inverted microscope equipped with 10×/0.25 Ph1 ACHROPLAN and 20×/0.8 Plan-APOCHROMAT objective lenses. DAPI and rhodamine-phalloidin were excited simultaneously with laser light of 405 and 555 nm, respectively. Fluorescence emission was detected through a 490 nm or 555 nm short-pass filter for DAPI and a 560 nm long-pass filter for rhodamine-phalloidin. Cell geometry in selected confocal z-slices was analyzed using Fiji ImageJ software (Schindelin et al., 2012). Images were processed with a median filter of radius 2.0 pixels to smooth while preserving edges, then with an unsharp mask with a radius of 1.0 pixels and weight of 0.9 to enhance phalloidin staining. The images were binarized according to local thresholding by the Sauvola method (Sauvola and Pietikainen, 2000) with a radius of 15 pixels to handle staining variations. The binary images were subjected to Watershed segmentation (Vincent and Soille, 1991) to separate joined cells and then area and perimeter were measured with Analyze Particles (Fiji Image J). Measured objects were compared with original images to omit non-cellular objects. We used hand-counting for sidedness as described (Gibson et al., 2006).

### Statistical analyses

Data were analyzed using SPSS Statistics 18.0 (IBM®). Sample sizes were calculated with Cohen's d tables or Mead's resource equation. Fisher's exact or Chi-squared testing was used for categorical data. Continuous data were analyzed for normality using Kolmogorov–Smirnov and Shapiro-Wilk tests and homogeneity of variance using Levene's test. Normally distributed data with similar variances were compared using an unpaired student *t*-test or one-way ANOVA followed by the Bonferroni multiple comparisons test. Non-parametric data were compared using the Mann–Whitney *U* test. Statistical significance was defined as *P*<0.05.
